# Metastatic Colorectal Cancer in the Era of Personalized Medicine: A More Tailored Approach to Systemic Therapy

**DOI:** 10.1155/2018/9450754

**Published:** 2018-11-05

**Authors:** Irene S. Yu, Winson Y. Cheung

**Affiliations:** ^1^University of British Columbia, Vancouver, Canada; ^2^University of Calgary, Calgary, Canada

## Abstract

Colorectal cancer is the second most common malignancy diagnosed in Canada. Despite declining incidence and mortality rates in recent years, there is still a significant number of cases that are metastatic at presentation. Fluoropyrimidine-based chemotherapy was the backbone of colorectal cancer treatment, but the addition of irinotecan and oxaliplatin to form combination regimens has significantly improved overall survival. In the past decade, the development of novel biologic agents including therapies directed against vascular endothelial growth factor and epidermal growth factor receptor has further altered the landscape of metastatic colorectal cancer treatment. However, clinical trials have demonstrated that not all patients respond to these therapies similarly and consideration must be given to individual patient- and tumor-related factors. A more tailored and biomarker driven approach to treatment selection can optimize outcomes and avoid unnecessary adverse effects. In this review article, we offer a comprehensive overview of the panel of clinical- and tumor-associated characteristics that influence treatment decisions in metastatic colorectal cancer and how this sets the foundation for a more personalized treatment strategy in oncology.

## 1. Introduction

Colorectal cancer (CRC) is the second most common malignancy in Canada with an estimated 26,800 new cases per year [1]. Approximately one-fifth of CRC cases are metastatic at the time of diagnosis [2]; a small proportion of these are potential candidates for curative metastasectomy or conversion therapy, but these are usually applicable to those with liver- or lung-limited metastases only. The remainder of metastatic CRC (mCRC) patients pursue systemic treatments, which have evolved significantly over the past 10 to 15 years. With best supportive care (BSC) alone, median overall survival (mOS) is approximately five months [3]. In the modern era of combination chemotherapy and newer biologic agents, overall survival can be extended to 2 years and longer [4]. There are growing efforts to personalize the treatment of mCRC so that appropriate subsets of patients are selected for specific therapies, with the goal of maximizing response and avoiding exposure to adverse effects. In this article, we summarize the most recent evidence on tumor- and patient-related factors that should be considered when selecting treatment options for patients with mCRC.

## 2. Evolution of Metastatic Colorectal Cancer Treatment

### 2.1. Chemotherapy Backbone

In the 1990s, 5-FU/leucovorin was the standard of care for treatment of mCRC, which resulted in mOS of up to 12 months [5, 6]. In 2000, Saltz et al. showed that the addition of irinotecan (IFL regimen) extended mOS by an additional 2 months when compared to 5-FU/leucovorin alone (14.8 versus 12.6 months, p=0.04) [7]. Subsequently, the Intergroup N9741 trial demonstrated superiority of FOLFOX over IFL, with mOS reaching close to 20 months (19.5 versus 15.0 months, p=0.0001) [8]. Due to the unfavourable toxicity profile of IFL, the FOLFIRI regimen was developed and found to have comparable survival outcomes to FOLFOX as evidenced in the GERCOR and GOIM trials [9, 10]. Thus, FOLFOX and FOLFIRI emerged as the new standard first-line chemotherapy options for the treatment of mCRC.

### 2.2. Addition of Biologic Agents

The development of biologic agents, namely, inhibitors of vascular endothelial growth factor (VEGF) and epidermal growth factor receptor (EGFR), began to further alter the landscape of mCRC treatment. The AVF 2107 study showed that the addition of bevacizumab to IFL resulted in significantly longer mOS (20.3 versus 15.6 months, p<0.001) [11]. The Intergroup N9741 trial was published in the same year and prompted many clinicians to add bevacizumab to the FOLFOX chemotherapy backbone. A pooled analysis by Hurwitz et al. confirmed that bevacizumab plus chemotherapy compared to chemotherapy alone resulted in modest improvements in mOS (18.7 versus 16.1 months, p=0.0003) and median progression free survival (mPFS) (8.8 versus 6.4 months, p<0.0001) [12]. The PRIME study showed that panitumumab, when added to FOLFOX, increased mPFS (9.6 versus 8.0 months, p=0.02) with a trend towards improved mOS (23.9 versus 19.7 months, p=0.072) in patients with KRAS wild-type (WT) tumors [13]. The addition of cetuximab to FOLFIRI yielded similar results in the CRYSTAL study, with longer mPFS (9.9 versus 8.4 months, p=0.0012) and mOS (23.5 versus 20.0 months, p=0.0093) [14]. Because of these previous studies, first-line therapy at the present time typically consists of either FOLFIRI or FOLFOX in combination with a biologic agent that targets either VEGF or EGFR.

More recently, further treatment options have been approved by the FDA, including aflibercept, ramucirumab, and regorafenib. These agents all work via antiangiogenesis mechanisms; they are generally reserved for use in the second line or later settings and provide only modest survival benefits of approximately 6 weeks [15–17]. Trifluridine-tipiracil (TAS-102) is an oral chemotherapy agent that was recently evaluated in the RECOURSE study among patients who were considered refractory or intolerant to conventional chemotherapy and biologic agents. The administration of TAS-102 was associated with a two-month prolongation in mOS (7.1 versus 5.3 months, p<0.001) [18]. This drug is already available for use in some Western countries. However, there is limited evidence thus far to guide clinicians on how best to select patients for these therapies or how best to sequence these available treatments. Predictive markers are greatly needed and represent an area of active research.

## 3. KRAS Status: Predicting Response to Anti-EGFR Agents

KRAS, also known as Kirsten Rat Sarcoma Viral oncogene homolog, is a gene located on human chromosome 12 that encodes for the KRAS protein, a GTP/GDP-binding protein involved in intracellular signal transduction in the EGFR pathway [19]. Activating KRAS mutations have been described in multiple malignancies, with downstream effects that include cell proliferation, antiapoptosis, and angiogenesis. KRAS mutations are found in more than 40% of CRC cases [20, 21], with point mutations involving exon 2 (codons 12 and 13) comprising about 95% of mutations [22]. However, more than 5,000 different KRAS mutations have been described in CRC tumors in the literature and the significance of these variants are less clear.

Cetuximab (Erbitux) is a recombinant human-mouse chimeric monoclonal antibody initially approved in 2004 for the treatment of EGFR-expressing mCRC tumors. It works via competitively binding to the extracellular domain of EGFR, to block phosphorylation and activation of downstream kinases. Early studies evaluated the role of anti-EGFR agents as add-on therapies when progression or intolerance to standard chemotherapy occurred. In the initial study by Cunningham et al., 329 irinotecan-refractory mCRC patients were randomized to cetuximab plus irinotecan versus cetuximab alone [23]. Response rates (22.9% versus 10.8%, p=0.007) and time to progression (4.1 versus 1.5 months, p<0.001) were significantly higher in the combination group, although mOS did not reach significance (8.6 versus 6.9 months, p=0.48). The benefit of cetuximab monotherapy was confirmed in the CO.17 trial by Jonker et al., which enrolled 572 pretreated patients and randomized them to receive either cetuximab or best supportive care (BSC) [24]. Cetuximab was associated with an improvement in PFS (HR 0.68, 95% CI 0.57 to 0.80, p<0.001) and OS (HR 0.77, 95% CI 0.64-0.92, p=0.005). Median survival was 6.1 months for the cetuximab group compared to 4.6 months for BSC.

Importantly, retrospective studies began to report an association between KRAS-WT status and EGFR therapy responsiveness [25, 26]. Karapetis et al. examined for KRAS exon 2 activating mutations in tumor samples from the CO.17 trial and found the benefit of cetuximab was restricted to the KRAS-WT group only [20]. With cetuximab, KRAS-WT patients experienced better mOS (9.5 versus 4.8 months, HR 0.55, 95% 0.41-0.74, p<0.001) and mPFS (3.7 versus 1.9 months, HR 0.40, 95% CI 0.30-0.54, p<0.001) compared to BSC. The KRAS-mutant (MT) cohort did not benefit from cetuximab with regard to PFS (HR 0.99, p=0.96) and OS (HR 0.98, p=0.89).

The role of cetuximab as part of first-line therapy was evaluated by Van Cutsem et al. in the CRYSTAL study [27]. A total of 1198 patients were randomized to compare FOLFIRI versus FOLFIRI plus cetuximab. A subsequent analysis had KRAS status available for 89% of patients from the original study. For KRAS-WT patients, FOLFIRI plus cetuximab significantly prolonged mOS (23.5 versus 20.0 months, HR 0.796, p=0.0093) and mPFS (9.9 versus 8.4 months, HR 0.696, p=0.0012) compared to FOLFIRI alone [14]. This benefit with cetuximab was not seen in KRAS-MT tumors in relation to PFS or OS.

Panitumumab (Vectibix) is a recombinant fully human monoclonal antibody approved in 2006 for EGFR-expressing mCRC after failing conventional chemotherapy regimens. Van Cutsem et al. randomized 463 patients who had progressed on standard chemotherapy to panitumumab versus BSC [28]. Panitumumab significantly prolonged PFS (HR 0.54, 95% CI 0.44-0.66, p<0.0001) but did not affect OS (HR 1.00, 95% 0.82 to 122). Amado et al. reanalyzed the data after obtaining KRAS status in 92% of the tumor samples [21]. Similar to cetuximab, panitumumab efficacy was restricted to KRAS-WT tumors. Treatment effect of panitumumab on PFS was significantly greater in the KRAS-WT (HR 0.45, 95% CI 0.34-0.59) compared to the KRAS-MT group (HR 0.99, 95% 0.73-1.36). Furthermore, 17% and 34% of the KRAS-WT group showed partial response and stable disease, respectively. In contrast, 0% and 12% in the KRAS-MT group experienced partial response and stable disease.

Panitumumab was further studied in the first-line setting in combination with chemotherapy. In the PRIME study, panitumumab was added to FOLFOX. KRAS results were available for 93% of the study participants. In the KRAS-WT cohort, panitumumab plus FOLFOX improved mPFS compared to FOLFOX alone (9.6 versus 8.0 months, HR 0.80, 95% CI 0.66-0.97, p=0.02) with a nonsignificant increase in OS [13]. In contrast, the KRAS-MT group had significantly reduced PFS (HR 1.29, 95% CI 1.04-1.62, p=0.02) and a trend towards worse mOS (15.5 versus 19.3 months, HR 1.24, 95% CI 0.98-1.57, p=0.068) with combination therapy versus FOLFOX alone. In a follow-up extension study, mOS reached significance at a median follow-up of 80 weeks, favoring panitumumab/FOLFOX for KRAS-WT tumors (HR=0.83, 95% CI 0.70-0.98, p=0.03) [29].

Most studies have focused on testing for KRAS mutations in exon 2, but KRAS-MT status in exon 2 alone does not consistently imply lack of response to anti-EGFR agents. Literature has suggested that other RAS mutations may also predict resistance to cetuximab and panitumumab [30, 31], including other exons of KRAS and NRAS. This has been reflected in the American Society of Clinical Oncology (ASCO) recommendations, which suggest the need for extended RAS testing prior to starting anti-EGFR therapy [32]. Recently, the FDA approved an extended RAS panel which detects 56 specific mutations in exons 2, 3, and 4 of both KRAS and NRAS genes. With respect to the optimal tissue used for testing, samples from the primary tumor and the metastatic site have shown good concordance for KRAS mutations, but evidence does not support reliable concordance rates for lymph node metastases or recurrent tumors [33, 34]. The recent development of circulating tumor DNA tests, or liquid biopsies, for the detection of KRAS mutants has shown variable concordance rates. Thus, these methods require further validation prior to widespread adoption and implementation [35, 36].

## 4. Impact of Primary Tumor Sidedness

It is known that tumors originating from different areas of the colon have distinct clinical and molecular characteristics. From an embryonic standpoint, the midgut gives rise to the right colon whereas the hindgut transforms into the left colon. This has epidemiological, pathological, and prognostic implications, but there has been emerging evidence to indicate that right- and left-sided tumors should also be treated differently. Most studies define left-sided (LS) tumors as those affecting the splenic flexure and areas distal to it, including the rectum [37], whereas right-sided (RS) tumors are proximal to the splenic flexure. Approximately two-thirds of CRC occur on the left side, with the remaining one-third affecting the right side [38]. RS cancers generally affect older patients and females, and the disease tends to be more advanced, poorly differentiated, and of mucinous pathology [39–41]. These RS (or proximal) tumors are also more likely to be associated with microsatellite instability, RAS/BRAF mutations, and CpG island methylator phenotype (CIMP)-high status [42]. In contrast, LS (or distal) tumors tend to carry frequent chromosomal instability and EGFR amplification. With respect to prognostication, RS tumors are associated with worse outcomes. A recent meta-analysis by Petrelli et al. looked at 1.4 million patients to determine the prognostic role of LS versus RS primary CRC localization; LS tumor location was associated with lower risk of death (HR 0.82, 95% CI 0.79-0.84, p<0.001) independent of disease stage [43].

New studies have reported that primary tumor localization (PTL) impacts specific treatment recommendations in the metastatic setting, specifically applicable to KRAS-WT patients [44, 45]. Holch et al. performed a meta-analysis and included 13 first-line trials to assess the prognostic and predictive role of RS versus LS CRC [46]. RS cancers were documented in 27% of cases, which is comparable to that reported in the literature. From a prognostic perspective, RS tumors were associated with worse PFS (HR 1.28, 95% CI 1.20-1.37, p<0.0001) and OS (HR 1.54, 95% CI 1.43-1.65, p<0.0001). The CRYSTAL and PRIME studies were analyzed together to see if PTL correlated with anti-EGFR therapy responsiveness. OS (HR 0.69, 95% CI 0.58-0.83, p<0.0001) and PFS (HR 0.65, 95% CI 0.54-0.79, p<0.0001) were significantly improved with first-line anti-EGFR therapy in the RAS-WT LS group, but not the RAS-WT RS cohort (OS HR 0.96, 95% CI 0.68-1.35, p=0.802 and PFS HR 0.82, 95% CI 0.57-1.19, p=0.307). Analysis of CALGB/SWOG 80405, FIRE-3, and PEAK studies evaluated the impact of both anti-EGFR and anti-VEGF agents. Similarly, LS tumors were associated with significantly improved OS (HR 0.71, 95% CI 0.58-0.85, p=0.0003) and a trend towards better PFS (HR 0.86, 95% CI 0.73-1.02, p=0.084) when treated with anti-EGFR therapy. Conversely, RS cancers were associated with significantly improved PFS (HR 1.53, 95% CI 1.16-2.01, p=0.003) and a trend towards improved OS (HR 1.3, 95% CI 0.97-1.74, p=0.081) with anti-VEGF agents.

These findings have altered practice with clinicians generally recommending the use of an anti-EGFR agent plus chemotherapy in the first-line setting in patients presenting with LS tumors that are RAS-WT, rather than bevacizumab. For RS tumors, there is a preference for bevacizumab combined with chemotherapy. We anticipate that major guidelines will be updated in the near future which will incorporate the use of PTL as a predictive marker for selecting therapy.

## 5. Microsatellite Instability: Its Role with Immunotherapy Agents

Microsatellite instability (MSI) results from a deficient mismatch repair system (dMMR), which is responsible for correcting nucleotide base mispairings that occur during DNA replication. The most commonly affected mismatch proteins include MLH1, MSH2, MSH6, and PMS2. MSI-high (MSI-H) status is present in 15% of CRC cases, with approximately 12% as sporadic cases and the remainder associated with Lynch Syndrome [47]. MSI-H tumors harbor excessive mutations and can generate “neoantigens” which can serve as a target for immunotherapy agents [48].

Pembrolizumab is an IgG4 monoclonal antibody against programmed death (PD-1) and was evaluated by Le et al. in a phase II study in which 53 mCRC patients with dMMR and proficient MMR (pMMR) were treated with pembrolizumab [49, 50]. Disease control rate, defined as complete response, partial response and stable disease, was seen in 89% (25/28) and 16% (4/25) in the dMMR and pMMR groups, respectively. Median PFS and OS were not reached for dMMR patients and were 2.4 and 6 months for pMMR cohort, respectively. The CheckMate 142 phase II trial assessed the efficacy of nivolumab, another PD-1 immune checkpoint inhibitor [51]. Overman et al. reported that of the 74 heavily pretreated patients, 31% (23/74) of patients had a documented objective response and 69% (51/74) achieved disease control > 12 weeks. Median duration of response has not been reached in the most recent publication, and 8 patients had disease response lasting greater than one year. Both pembrolizumab and nivolumab appear to provide durable benefit to responders.

Despite the small size of these studies, the FDA has approved pembrolizumab and nivolumab for use in MSI-H mCRC that has progressed on chemotherapy. This is reflected in the most recent National Comprehensive Cancer Network (NCCN) guidelines for metastatic MSI-H CRC [52]. However, the widespread use of these immunotherapy agents is currently limited because the number of metastatic cases that are MSI-H is low [53]. Further exploration into other pathologic and genetic factors beyond MSI status as predictive factors is required. We also await results from the phase III KEYNOTE-177 study that is examining the use of first-line pembrolizumab versus investigator's choice of chemotherapy in MSI-H mCRC patients [54].

## 6. BRAF: Prognostic and Possible Predictive Factor

The BRAF gene is located on human chromosome 7 and encodes the BRAF protein, also known as serine/threonine-protein kinase B-Raf. It is a member of the Raf family of protein kinases involved in cellular signal transduction, downstream of KRAS. Activating BRAF mutations mostly occur in codon 600 and is known as the V600E mutation; this is found in < 10% of sporadic CRC cases [55]. There is strong evidence for its use as a prognostic factor compared to its predictive value, although data are emerging with respect to predicting response to anti-EGFR therapy.

Tran et al. identified 524 mCRC patients with known BRAF status and evaluated the impact of BRAF mutation on prognosis [56]. BRAF mutants (BRAF-MT) were significantly associated with poorer survival with mOS of 10.4 versus 34.6 months (p<0.001). The BRAF-MT group was more often linked to RS tumors and microsatellite instability. BRAF mutants also displayed a distinct pattern of metastatic spread with higher rates of peritoneal and distant lymph node involvement. Subsequent analyses of phase III trials have confirmed that BRAF-MT are indicators of worse prognosis [14, 57]. In a pooled analysis by Venderbosch et al., greater than 3000 patient samples were examined [58]. Patients with BRAF-MT status had worse PFS (HR 1.34, 95% CI 1.17-1.54) and OS (HR 1.91, 95% CI 1.66-2.19) when compared to their BRAF-WT counterpart.

There is growing evidence that tumors harboring the BRAF V600E mutation are not as likely to respond to anti-EGFR therapies, although studies have been conflicting. A meta-analysis by Pietrantonio et al. evaluated both cetuximab and panitumumab in both first and nonfirst-line settings; the addition of EGFR agents did not increase the benefit of standard therapy in the BRAF-MT group [59]. PFS (HR 0.88, 95% CI 0.67-1.14, p=0.33) and OS (HR 0.91, 95% CI 0.62-1.34, p=0.63) were not significantly different compared to the control group. Conversely, Rowland et al. also conducted a meta-analysis of 8 trials and found longer OS in BRAF-WT patients with the use of anti-eGFR therapy (HR 0.81, 95% 0.70-0.95) which was not seen in BRAF-MT patients. However, the test for interaction was not statistically significant, so the authors concluded that the observation may have been secondary to chance and there is presently insufficient evidence to show that BRAF-MT cancers respond differently to anti-EGFR agents [60].

Despite the variable results, the ESMO guidelines have recommended against using cetuximab or panitumumab in patients harboring a BRAF V600E mutation [61]. Studies are underway to evaluate whether the addition of a BRAF inhibitor (e.g., vemurafenib) can help improve outcomes in BRAF-MT patients when used in combination with anti-EGFR therapy [62].

## 7. Elderly Population

Approximately 60% of CRC are diagnosed in patients who are aged greater than 65 years [1], with a median age of diagnosis of 67 years among all cases. The proportion of older adults has been steadily increasing over the past few decades and it is expected to comprise one-quarter of the North American population by 2036 [63]. Hence it is also anticipated that the number of mCRC cases in the elderly will increase in the near future. This subset of the population has special considerations, yet historically they have been underrepresented in most clinical trials, with only one-third of clinical trial participants aged ≥ 65 years [64]. In recent years, there has been increasing recognition of the need to place more emphasis on geriatric oncology in order to address the impending increase in cancer burden in this population.

With aging, there is decline in the function of critical organs. Changes to liver and renal physiology may slow drug metabolism and elimination, which can increase treatment toxicities through variable pharmacokinetics and pharmacodynamics; bone marrow reserve also diminishes and older adults are more susceptible to chemotherapy-related cytopenias [65]. The presence of significant comorbidities also increases with age; hypertension and diabetes are the most common ailments in an observational study of newly diagnosed cancer patients [66]. This not only impacts the overall frailty status of the individual, but also has implications for some of the systemic agents that are commonly utilized in mCRC.

Chronological age does not correlate well with functional status and oncologists often utilize other tools to assess functional status. In 2005, the International Society of Geriatric Oncology (SIOG) published recommendations on the use of geriatric assessments [67], but their use in practice has been limited because they are usually very resource-intensive. Geriatric assessments refer to multidisciplinary evaluations that include multiple domains, consisting of an examination of a patient's functional status, psychological health, polypharmacy, comorbidities, nutrition, social support, and cognition. Because adoption of full geriatric assessments has been variable, SIOG subsequently released an updated review of 17 shorter screening tools commonly used in older cancer patients [68], and the consensus statement deemed that the G8 tool was the most robust. Thus, it was recommended as a possible initial screening tool to identify patients in need of further evaluation by full geriatric assessments. Of note, the Eastern Cooperative Oncology Group (ECOG) and Karnofsky Performance Status (KPS) assessments are also commonly used by oncologists in the adult population, but their utility in the elderly is questionable. In one study, with ECOG score of 1 as the cut-off, the sensitivity and specificity were 94% and 55% respectively for predicting fitness for treatment in the elderly [69]. Likewise, KPS was compared to geriatric assessments in two studies, and showed extreme variability in sensitivity and specificity, ranging from 29-78% and 44-91%, respectively, for a KPS cut-off value of < 80 [69, 70]. Unfortunately, there is a lack of standardization for geriatric assessments. Moreover, insufficient personnel and resources in a busy clinical practice may limit the widespread acceptance of their use. In general, use of a screening tool is currently suggested and if abnormal, a more comprehensive geriatric assessment is still recommended.

For fit elderly patients, treatment of mCRC generally mirrors that of younger adults with first-line doublet chemotherapy (FOLFOX or FOLFIRI) with or without a biologic agent. Multiple studies have suggested that use of fluoropyrimidine-oxaliplatin-containing regimens in the elderly results in similar mOS, mPFS, and response rates when compared to the younger population [71–73], although findings across studies have not always been consistent. One study by Arkenau et al. showed that mOS was significantly shorter in those > 70 (18.8 versus 14.4 months, p=0.013) which persisted in their multivariate analysis that adjusted for comorbidities and other confounders [72]. Cen et al. performed a population based study with over 46,000 older patients and found that oxaliplatin-containing regimens have higher incidences of adverse effects, including nausea, neutropenia, and neuropathy when compared to 5-FU alone [74]. With respect to FOLFIRI, evidence is also discordant but it appears to suggest comparable safety and efficacy in older and younger patients [75–77].

In moderately fit patients, it is reasonable to treat with a fluoropyrimidine (5-FU preferably) with bevacizumab based on available literature. The MRC FOCUS2 trial showed that 5-FU plus oxaliplatin resulted in a trend for better mPFS (5.8 versus 4.5 months, p=0.07) compared to 5-FU alone [78]. This trial was initiated at 80% of standard dosing, with the option of escalating the dose if the patient tolerated initial treatment. Based on the FFCD 2001-02 trial, investigators proposed trying infusional 5-FU based therapy alone as the addition of irinotecan did not significantly increase PFS or OS [77]. The AVEX trial by Cunningham et al. was a phase III trial which enrolled patients > 70 years only [79]. Patients received either bevacizumab plus capecitabine or capecitabine alone; the combination resulted in longer mPFS (9.1 versus 5.1 months, p<0.0001). However, one caveat is that capecitabine was associated with more treatment-related adverse effects versus short-term infusional 5-FU, despite the convenience of oral dosing. Bevacizumab must also be prescribed with caution as the risk of arterial thromboembolism is high in elderly patients, and clinicians should avoid its use in those with recent myocardial infarction, cerebrovascular accident, or severe uncontrolled hypertension.

For elderly patients with poor functional status, options include single agent fluoropyrimidine with 5-FU or capecitabine or an anti-EGFR inhibitor if they are KRAS-WT. In a retrospective analysis, Crosara Teixeira et al. examined the effects of chemotherapy on those with poor functional status (i.e., ECOG 3/4) [80]. When stratified by ECOG status, chemotherapy led to nonsignificant survival gain (6.8 versus 2.3 months, p=0.13) but sample size was limited with only 240 patients. For those with better performance status (i.e., ECOG 2), a pooled study of 9 trials by Sargent et al. showed that ECOG 2 patients derived similar benefit compared to those with ECOG 0/1, but there were more adverse effects and higher 60-day mortality (2.8 versus 12%, p<0.0001) in those with worse ECOG status [81]. However, the 5-FU bolus was typically omitted and the “stop and go” approach was also adopted by clinicians.

## 8. Diabetes: Special Considerations

Diabetes mellitus (DM) is a growing problem, with an estimated 10 million Canadians living with prediabetes or diabetes [82]. The prevalence is expected to increase by over 40% by 2025. DM itself is considered a moderate risk factor for CRC, with an estimated relative risk of 1.38 and 1.20 for colon and rectal malignancies, respectively, when compared to nondiabetics [83]. At this time, however, there are no specific surveillance recommendations for this particular group. Nevertheless, special considerations must be taken when treating mCRC patients who also have coexisting DM, as there are implications for their systemic treatment.

Up to 50% of diabetic patients have evidence of peripheral neuropathy [84], most typically manifesting as “stocking-glove” sensory loss affecting the longer axons preferentially. Thus, the use of oxaliplatin as a part of FOLFOX must be considered carefully. Oxaliplatin is associated with two distinct neurotoxicity syndromes, including acute neurotoxicity and chronic cumulative sensory neuropathy. The mechanism for the latter is hypothesized to be the entry of oxaliplatin into the dorsal root ganglion, leading to the apoptosis of neurons [85]. However, there has been conflicting evidence on the influence of DM on the incidence on oxaliplatin-induced neurotoxicity. In a retrospective pooled analysis of both adjuvant and metastatic CRC cases, Ramanathan et al. examined whether the diagnosis of DM affected the incidence and severity of peripheral neuropathy after oxaliplatin [86]. A total of 1587 patients were included and 8.5% had DM at baseline prior to oxaliplatin therapy; patients with preexisting peripheral neuropathy greater than grade 1 were excluded from the study. Overall, the incidence of grade 1, 2, and 3 peripheral neuropathy for patients with and without DM were similar (46.7% versus 45.0%; 26.7% versus 28.6%; 12.6% versus 13.0%, respectively). The authors therefore concluded that the presence of DM was not associated with an increased risk of peripheral neuropathy. Conversely, Ottaiano et al. examined 102 stage II/III CRC patients treated with capecitabine and oxaliplatin (CAPOX) adjuvant therapy and found a significant association between DM and the occurrence of chronic neurotoxicity (47.3% DM patients versus 1.2% non-DM patients, p<0.0001), but this study was limited by a small sample size (n=19 for DM) and the duration of chronic neuropathy was not clearly defined [87].

Studies have shown that the long-term effect of oxaliplatin on peripheral neuropathy is dose-related, with persistent neuropathy seen in 10-15% of patients after a cumulative dose greater than 780-850 mg/m2 [88]. In a retrospective study by Uwah et al., the presence of DM did not impact the severity of oxaliplatin-induced peripheral neuropathy but data suggested that DM patients may develop the complication at a lower mean cumulative dose of oxaliplatin compared to those without DM (388 mg/m2 versus 610 mg/m2) [89]. Furthermore, the time for 50% of patients to develop neuropathy was earlier for the DM group compared to the non-DM group (5th versus 8th cycle, p=0.35), although this was not statistically significant.

Individuals with DM may also experience frequent diarrhea, described in up to one-quarter of diabetics. This may be related to multiple mechanisms, including disordered motility due to diabetic autonomic neuropathy and/or diabetic medications such as metformin. There are limited data on the risk of diarrhea in DM patients receiving treatment for CRC. A cohort study by Meyerhardt et al. evaluated the impact of DM on chemotherapy-associated toxicity in stage II/III CRC and found that DM patients have an increase risk of treatment-related diarrhea when compared to non-DM individuals [90]. Hence, clinicians must educate patients about the risks of irinotecan, which frequently causes diarrhea.

One aspect that may be neglected while caring for CRC patients with DM is the risk of side effects from premedications, specifically dexamethasone which is commonly included as an antiemetic prior to and after receiving chemotherapy. Glucocorticoids affect glucose homeostasis via downregulation of glucose transporters in skeletal muscle, increased hepatic production of glucose, inhibition of insulin binding to its receptors on cellular surfaces, and decreased insulin secretion from islet cells [91]. Therefore, DM patients may experience high blood glucose levels when taking dexamethasone. Nonetheless, there are currently no guidelines supporting modified antiemetics for DM patients. In practice, oncologists should ask patients to monitor for hyperglycemia and adjust their antidiabetic medications accordingly, if required.

Overall, published literature does not appear to endorse a different treatment approach for CRC patients with or without DM [92]. The most concerning clinical issue is peripheral neuropathy, but based on limited data, preexisting DM does not appear to affect the incidence or severity of oxaliplatin-induced neuropathy. There is a suggestion that it may occur with lower cumulative doses and with fewer cycles. For the time being, clinicians should be particularly cognizant of this toxicity in their DM patients, and may need to discontinue oxaliplatin at an earlier point in the treatment trajectory in order to prevent long-term debilitating effects.

## 9. Screening and Surveillance Strategies

The most common strategies utilized in colorectal cancer screening include guaiac-based fecal occult blood test (FOBT), fecal immunochemical test (FIT), endoscopy, and CT colonography. Major guidelines vary slightly in their recommendations, but screening is generally recommended in those with average risk between the age of 50 to 75 years using FIT or an endoscopic procedure [93, 94]. Clinically, the FIT test is rapidly replacing the use of FOBT with the capability to detect human-specific globin. In a large meta-analysis by Wieten et al., the use of FIT was associated with less interval colorectal cancer diagnosed after a negative test compared to FOBT (pooled incidence rates of 20 versus 34 per 100,000 person-years) [95]. The optimal interval between time from a positive fecal test to colonoscopy is unclear, but a large cohort study of over 70,000 patients with positive test results showed that a delay beyond 10 months led to a higher risk of stage IV disease (OR 2.71, 95% CI 1.06-6.89) [96]. This risk was not seen in those who underwent a colonoscopy between 7 to 9 months after a positive fecal test. Thus, these data support a timely referral for endoscopic examination of the colon after a positive FIT test.

In those patients who are cured of their colon cancer, approximately 30% of stage I-III and up to 65% of stage IV patients will recur [97]. The recent COLOFOL trial showed that low intensity surveillance (CEA and CT scans at 12 and 36 months only) in over 2,500 patients with resected stage II and II disease was comparable to conventional surveillance strategies, in terms of frequency of recurrence detection, 5-year survival, and cancer-specific mortality [98]. Currently, most guidelines recommend CEA every 3 to 6 months along with a CT annually for the first 3 to 5 years [97]. With the emerging data, it is anticipated that these may be updated to reflect less frequent testing. For resected stage IV disease, ESMO and NCCN guidelines recommend more intensive imaging, with CT scans every 3 to 6 months for 2 to 3 years [52, 99]; given the heterogeneity of this population, surveillance is often individualized in practice.

## 10. Future Directions

As interest in the use of predictive clinical and genetic biomarkers continues to increase, human epidermal growth factor 2 (HER2) presents as an attractive target for further development. The HER2 oncogene encodes for a transmembrane glycoprotein receptor, which has a critical role in intracellular signal transduction pathways involved in cell growth, cell differentiation, and angiogenesis [100]. Its role in the pathogenesis of breast cancer is well-established and the success of trastuzumab has significantly impacted the treatment of HER2-positive breast cancer [101]. A small proportion of CRC also overexpresses HER2, which can be detected through immunohistochemical staining, in situ hybridization for gene amplification, or polymerase chain reaction for RNA overexpression. In the phase II HERACLES trial, 914 KRAS-WT mCRC patients were screened for HER2 positivity; 48 (5%) patients were identified as HER2+ and 27 patients were eligible to receive trastuzumab and lapatinib. After a median follow-up of 94 weeks, 30% (8/27) patients had an objective response and 44% (12/27) achieved stable disease [102]. In the recent phase IIa multiple basket MyPathway study, trastuzumab and pertuzumab achieved 38% (14/37, 95% CI 0.23-0.55) objective response rates [103]. It is anticipated that further studies will attempt to clarify the promising role of HER2+ targeted agents, although widespread use may be limited by the small proportion of mCRC that overexpress the oncogene.

There is significant interest in looking at different targets involved in the mitogen-activated protein kinase (MAPK) pathway, which is responsible for regulating fundamental cellular processes such as growth, proliferation, differentiation, migration and apoptosis [104]. The pathway consists of multiple signalling molecules including RAS, RAF, MEK, and ERK; abnormalities along this pathway play a critical role in oncogenesis and hence present as potential targets for therapy. It was hypothesized that MEK inhibitors may work in synergy with PD-L1 inhibitors by upregulating MHC class 1 expression to increase antigen presentation on the surface of tumor cells for recognition by T cells [105]. The MEK inhibitor cobimetinib was studied in combination with the PD-L1 inhibitor atezolizumab in the phase III trial IMblaze370 consisting of 363 metastatic colorectal cancer patients, 91.7% of whom had MSS or MSI-low tumors [106]. However, it did not meet its primary endpoint for OS when comparing it versus regorafenib (8.9 versus 8.5 months, HR 1.0, 95% CI 0.73-1.38). There is an ongoing phase II study of BRAF/MEK inhibition in combination with PD-L1 blockade in BRAF V600E mutants, which can provide insight into another subset of this population [107].

Results are more promising for BRAF/MEK inhibitors in combination with anti-EGFR therapy in the treatment of BRAF V600E mutated cases. The combination of BRAF/MEK inhibitors have been shown to extend PFS and OS in BRAF-mutated metastatic melanoma [108] but melanoma cells express low levels of EGFR activity. The benefit of utilizing BRAF inhibitors does not apply to colorectal cancer due to the rapid feedback activation of EGFR [109]. The phase III BEACON trial is currently recruiting 640 patients to study the efficacy of combination BRAF inhibitor encorafenib, MEK inhibitor binimetinib and anti-EGFR agent cetuximab. Safety lead-in results were presented at GI ASCO, which included 30 patients with an overall response rate of 41%, with tumor regression observed in all but one patient [110]. Updated data at ESMO showed PFS of 8 months, with OS not yet reached [111]. Subsequently, the FDA has granted breakthrough therapy designation for the triplet therapy.

## 11. Conclusion

As the second most common malignancy in the developed world, the burden of CRC will continue to grow, especially with the aging population. Despite the implementation of routine CRC screening at the population level, a substantial proportion of CRC are still diagnosed at the metastatic stage. Fortunately, there has been significant progress in the development of new targeted therapies in the past decade. When used as monotherapy or in combination with preexisting chemotherapy, improvements in survival have been achieved. Currently, one of the most pressing issues is finding more effective ways to leverage these advances by selecting the right patients for the right therapy at the right time. Tailoring existing therapies based on tumor sidedness, KRAS, MSI, or BRAF status as well as individualizing treatments for elderly or DM patients represent our current efforts to provide more personalized care in oncology. However, these likely reflect only initial steps and more work is clearly warranted on furthering our understanding on additional clinical and pathological characteristics that can be used to personalize treatment in CRC.

## Data Availability

Data will be made available upon request.
